# Bold personality makes domestic dogs entering a shelter less vulnerable to diseases

**DOI:** 10.1371/journal.pone.0193794

**Published:** 2018-03-29

**Authors:** Sara Corsetti, Simona Borruso, Mario Di Traglia, Olga Lai, Lavinia Alfieri, Agnese Villavecchia, Giuseppe Cariola, Alessandra Spaziani, Eugenia Natoli

**Affiliations:** 1 Biology and Biotechnology Department, University of Rome *Sapienza*, Rome, Italy; 2 Public Health and Infectious Diseases, University of Rome *Sapienza*, Rome, Italy; 3 Experimental Zoo-prophylactic Institute Latium and Tuscany, Rome, Italy; 4 Canine consultant, free-lance, Rome, Italy; 5 Interzonal Dog Shelter, Local Health Unit Rome 3, Rome, Italy; University of Bari, ITALY

## Abstract

It is widely recognised that for vertebrate species, personalities vary along an axis with extremes represented by ‘proactive’ and ‘reactive‘ individuals. The aim of this study was to verify whether there is a relationship between personality and disease vulnerability in domestic dogs (*Canis familiaris*) exposed to an intensely stressful situation such as entering a shelter. Twenty-eight shelter dogs participated in the study. The ethogram consisted of approximately 100 behavioural patterns. Behavioural observations of dogs in their new environment, a Novel Object and a T-maze test were used to evaluate the personality of the dogs captured as strays and entering the shelter. A blood sample from each dog was obtained at admission into the shelter and after a month to evaluate their immunological state. Based on PCA analyses of observational combined with experimental data, the dogs were ordered along the boldness-shyness axis, with the first being the boldest. Excluding one (the 6th), the first 10 dogs showed an improved health status: absence of disease symptoms during the 30 days of monitoring and improved immunological parameters; the opposite was found for shy dogs. The results of this research seem to confirm findings in other vertebrate species, i.e., bold and shy dog vulnerability to diseases might be different, especially when they must cope with a stressful and highly infectious environment such as a dog shelter.

## Introduction

For vertebrate species, it is widely recognised that personalities vary in the way they cope with stress: along an axis, the extremes of which are represented by ‘proactive’ and ‘reactive’ responses [1] individuals are defined as ‘bold’ and ‘shy‘, respectively [2]. Proactive individuals are generally bold, tend to explore quickly and try to manipulate the situation even in a new context. Thus, on the one hand, they cannot be defined as “neophobic” [1]. Consequently, they are more prone to disperse. For this reason, the Novel Object test has been used in animal personality testing and, more specifically, as a way to measure the ‘proactive’ and ‘reactive’ axis, in many species (e.g., barnacle goose (*Branta leucopsis*), [3]; warbler (spp.) [4]; working dogs, [5]; pigs (*Sus scrofa*) [6–7]; and the rainbow trout *(Oncorhynchus mykiss)*, [8–9]). On the other hand, bold individuals have been found to be less prone to make changes in established procedures or to alter their routine [1, 10–11].

Conversely, reactive individuals are generally shy, tend to be more cautious and are more sensitive to external stimuli. They are slower in taking decisions and they tend and try to adapt themselves to the situation more than to manipulate it. Although this tendency makes them less exposed to the risks of being injured or killed or infected by a virus (in all the cases in which the transmission necessitates a contact between saliva and blood, that can occur during fights, see for example [12]), empirical data demonstrate that shy individuals show higher hypothalamic-pituitary-adrenal axis reactivity in response to stress than bold individuals (e.g., [1, 13–17]), resulting in an increase in circulating glucocorticoids [18]. If the stressor persists for some time, shy individuals become more vulnerable than bold ones to stress-related disorders.

It has been extensively demonstrated that, for domestic dogs (*Canis familiaris*), entering in a shelter represents a stressful event [19–27]. Spatial and social restriction, exposure to novel environments and eventual separation from an attachment figure are some of the stressful events faced by a dog in this situation [28].

Therefore, the aim of this study was to examine whether there is a relationship between personality and disease vulnerability in domestic dogs entering a shelter. In particular, we hypothesised that boldness (as a composition of active, attentive, confident and social traits vs anxious and subordinate traits) [1, 29–32] might be the most important personality dimension relevant to coping with stressful shelter conditions and that animals with a higher boldness rating would be more likely to possess the skills to cope with this challenging situation. We predicted, therefore, that compared with shy animals, bold animals would display different levels of specific physiological parameters linked to their immunological profile and a reduced risk of contracting the most common diseases.

Confirmation of such a hypothesis could help dog shelter management improve the welfare of dogs in the future.

To assess the personalities of dogs entering a shelter, we used both observational measures of their spontaneous behaviour in their novel environment and two experimental paradigms: the novel object and the T-maze test. Indeed, studies have shown that the best characterisation of the temperament of dogs is obtained from an assessment of their behaviour in multiple contexts [33–34].

## Materials and methods

### Animals and housing

The subjects of this study were 28 domestic dogs (20 males and 8 females) aged between 1 and 7 years (estimated by veterinary standard methods, i.e., evaluation of teeth consumption, status of fur, among others) that were captured as strays, of which 20 were mixed-breed and 8 were clearly purebred-derived dogs (German shepherd, Maremma sheep dog, Pitbull, Rottweiler, Cavalier King and Belgian shepherd). Three age classes were identified: 1^st^ from 1 year to 2 years and 11 months (15 dogs); 2^nd^ from 3 to 5 years (9 dogs); and 3^rd^ from 6 to 7 years (4 dogs). The dogs had to be in good health condition and negative for *Leishmania infantum* and *Ehrlichia canis* upon entry into the dog shelter. Another 24 dogs were excluded because they were unhealthy at entry (7 dogs), too aggressive (3 dogs), pregnant (1 dog), rescued by owners (8 dogs), able to climb the fence of the fenced area where the tests were conducted (1 dog) or adopted during the observational period (4 dogs).

The study was carried out at the Municipal Interzonal Dog Shelter "La Muratella" in Rome. The dogs were housed alone or in pairs in cages (4 m^2^) with indoor and outdoor zones. They were fed early in the morning and late in the afternoon; the standard food provided in the shelter is both dry and wet dog food of high quality, and each dog receives an adequate quantity for its size and age. The cages were cleaned twice a day, just before food distribution. Each dog was taken out of its cage daily by the staff and/or volunteers for a walk in a fenced area inside the shelter.

### Dog health monitoring

The protocol provided in the dog shelter "La Muratella", where the study was carried out, required that a dog, upon entering the shelter, was subjected to a clinical examination before housing. Thus, each dog captured as a stray entering the shelter was subjected to a clinical examination during which, among other routine checks (i.e., temperature control, visual physical examination of the ears and mouth), the animal was checked and treated for ecto- and endo-parasites. If judged in good health, it was selected for this study. In Italy, it is enforced by law a collection of blood sample (between 6 to 8 cc, from the brachial vein) of the dog entering a shelter before housing it, in order to check if the animal is positive for *Leishmania infantum*. In this study, several other parameters (see below) were quantified from the same blood sample. None of the selected dogs necessitated anaesthesia to keep them quiet during blood sampling. Afterwards, during the same clinical visit, each selected dog was vaccinated against distemper, Rubbarth’s hepatitis, leptospirosis and diseases due to paramyxovirus and to parvovirus (gastroenteritis). All dogs were vaccinated in the same way, with a standard veterinary protocol. Once in the shelter, the health of each dog selected for this study was accurately monitored by a veterinarian who visited the dog under observation every day for one month; on the basis of a visual inspection and, if necessary, a physical examination, any symptom that eventually appeared (e.g., cough, diarrhoea, vomit, inflammations, otitis, among others) was registered. At the end of the 30 days of monitoring, a second blood sample was collected to evaluate the same parameters (see below), a routine that falls in good veterinary practices, allowed by the Italian National Laws, to monitor the health of the animals.

We selected a 30-day interval to monitor infectious diseases because the database of this shelter underlined that the incidence of diseases increases in that time and plateaus after 30 days.

### Behavioural observations

Each of the 28 dogs was observed in its home-cage for a total of 5 hours (1 hour/day for 5 consecutive days, where possible) from a distance that ranged from 2 to 5 m, within the first week of permanence in the shelter, during the daytime between 8.00 and 18.00 h. All dogs became rapidly accustomed to the presence of the observer since the latter did not interact with them, neither during the observation session nor between observation sessions: after a few minutes, they just went on performing their ordinary activities. The ethogram consisted of more than 100 behavioural patterns (Table 1, described previously by De Palma et al. [35]. Data were collected using the focal animal sampling method [36]. The observations were made with a check sheet recording of the selected behavioural patterns of one dog (the focal animal) with ‘All occurrences’ and ‘1/0’ methods (60 s interval) [36].

**Table 1:** The ethogram utilised in this study for the observational session in cages (De Palma et al. 2005).

**Activity:**
Standing: staying in an upright position, on four legs.
Walking: walking in the fenced area within the shelter.
Trotting: trotting in the fenced area within the shelter.
Galloping: galloping in the fenced area within the shelter.
In/out: going in and out of the indoor/outdoor zone of the cage.
**Aggressive behaviour:**
Growling: threatening vocalisation coming from the throat.
Sideways glance: looking transversely with the head upright or bent. The glance is threatening.
Raising fur: raising the fur of the head, body and tail so that the dog appears to have a larger size and is thus more threatening.
Curling lip: light raising of the upper lip, usually only on one side, with a threatening partial display of the teeth.
Showing teeth: curling of the upper and lower lips while opening the mouth with a threatening display of the teeth, particularly the canine teeth.
Dashing at bars: dashing at bars in the direction of the observer, of another person or of another dog.
**Displacing activities:**
Body shaking: shaking the body quickly sideward.
Scratching: raising one hind leg and vigorously scratching part of the body.
Muzzle licking: passing the tongue over the muzzle.
Yawning: opening the mouth and inhaling and exhaling air.
Spinning in place: turning on itself, when this behaviour is recorded in the cage, it might take the place of running.
Auto-grooming: cleaning itself with the tongue and the teeth.
**Stereotyped or repetitive behaviour:**
Repetitive pacing in circles: repetitive walking in a circle within the cage.
Licking or biting compulsively: repeatedly licking or biting the bars, the wall and objects.
Catching flies: trying to catch an imaginary fly with the mouth, clutching at empty air with the teeth.
Coprophagy.
Self-mutilation: licking itself continuously in same part of the body, so intensely to cause abrasions or even wounds.
**Attention:**
Raising ears
Looking outside: looking outside the cage.
Looking out carefully: looking outside the cage very carefully; the position resembles that described for "prompt" but the dog is not ready to spring up.
Looking at observer: looking at the observer.
Looking at unknown people: looking at people the dog does not know.
Looking at volunteer: looking at a shelter volunteer worker.
Looking at dog: looking at another dog.
Raising foreleg: raising one foreleg.
Raising forelegs on wall: raising both forelegs onto the wall or onto the bars, looking carefully outside
**Olfactory investigation**
Sniffing environment: putting the muzzle on the ground, on the wall, or on the objects in the cage, the dog sniffs the environment.
Sniffing air: raising the head, moving the nostrils and breathing the air to perceive odours.
Sniffing observer: pointing the muzzle towards the observer, the dog moves the nostrils clearly trying to perceive the odours of the observer.
Sniffing unknown people: pointing the muzzle towards people the dog does not know, the dog moves the nostrils clearly trying to perceive their odours.
Sniffing volunteer: pointing the muzzle towards volunteers working in the shelter, the dog moves the nostrils clearly trying to perceive their odours.
Sniffing dog: pointing the muzzle towards another dog, the subject moves the nostrils clearly trying to perceive the object’s odours.
**Dominant behaviour:**
Staring at: gazing at the observer, another person or another dog right in the eyes.
Stiff body and tail still: standing still in an upright posture, with the ears raised and turned forward, the four legs straight and rigid and the tail immobile and rigid at a medium height.
Raised tail: the tail is held high while it is still.
Wagging with the tail held high: moving the tail sideward while held high.
Pricked-up ears: holding the ears forwards while assuming an upright body posture with head and tail held high, legs straight and stiff.
Paw or a muzzle on a conspecific’s back: putting the muzzle or one forepaw or both over the back of another dog.
**Submissive behaviour:**
Avoiding eye contact: looking away from the observer, another human or another dog, who is looking at the subject.
Lowering head: lowering the head in front of the observer, another human or another dog.
Ears down: putting the ears down, pressed on the head, or holding them backwards.
Cringing: lying with the ventral region in contact with the ground.
Tail between the legs: holding the tail down or tightly between the hind legs and against the belly.
Lying down on back: laying down on the back exposing the ventral side of the chest and sometimes the abdomen.
**Vocal communicability:**
Barking: emitting an abrupt, loud, noisy, and often repetitive vocalisation characteristic of dogs.
Whining: emitting a mournful vocalisation.
Grumbling: emitting a low and deep vocalisation that seems to come from the chest, the dog generally has the mouth closed.
Mumbling: emitting a vocalisation that consists of a sort of inside murmur.
Howling: emitting a vocalisation that consists of a long, high and mournful sound; characteristic of wolves, quite rare in dogs.
Snorting: emitting a vocalisation while puffing out its cheek and emitting air.
**Affiliative behaviour:**
Waving tail: the tail is wagged sideward but not held high, in a relaxed manner.
Giving the foreleg: raising one of the forelegs and leaning it in the direction of the observer.
Leaning on bars: leaning the body in direct contact with the bars of the cage.
Licking the mouth: licking the muzzle of other dogs.
Passive contact: staying in contact with some part of the body, without sleeping.
Allo-grooming: cleaning the fur of another dog, licking and nibbling.
**Resting:**
Sitting: sitting down with the rump leaning on the ground.
Lying: lying down on the ground.
Dozing: curling up, the dog is half asleep.
**Playing:**
Inviting to play: inviting another dog or human to play: the dog bends down with the forelegs outstretched on the ground and the rump upwards, or brings an object, runs around and jumps.
Answering invitation to play: playing with another dog after having been invited to do so.
Showing object: taking an object into the mouth and showing it to another dog or to the observer.

### Novel object test

We placed an unknown object in a large outdoor enclosure (approximately 320 m^2^) and introduced one dog a time, allowing it to explore for 30 minutes. The dog was alone during the test and familiar with the enclosure. The unknown, novel object was a big, inflatable killer whale, which is usually utilised by children on the beach. This object was chosen because i) it had a strange and unknown shape that was not linked to threatening shapes written in the phylogenetic memory of the species (e.g., a bear); ii) it had large dimensions and a dark colour and thus was potentially intimidating; iii) it was plastic and was therefore washable.

**Fig 1:**
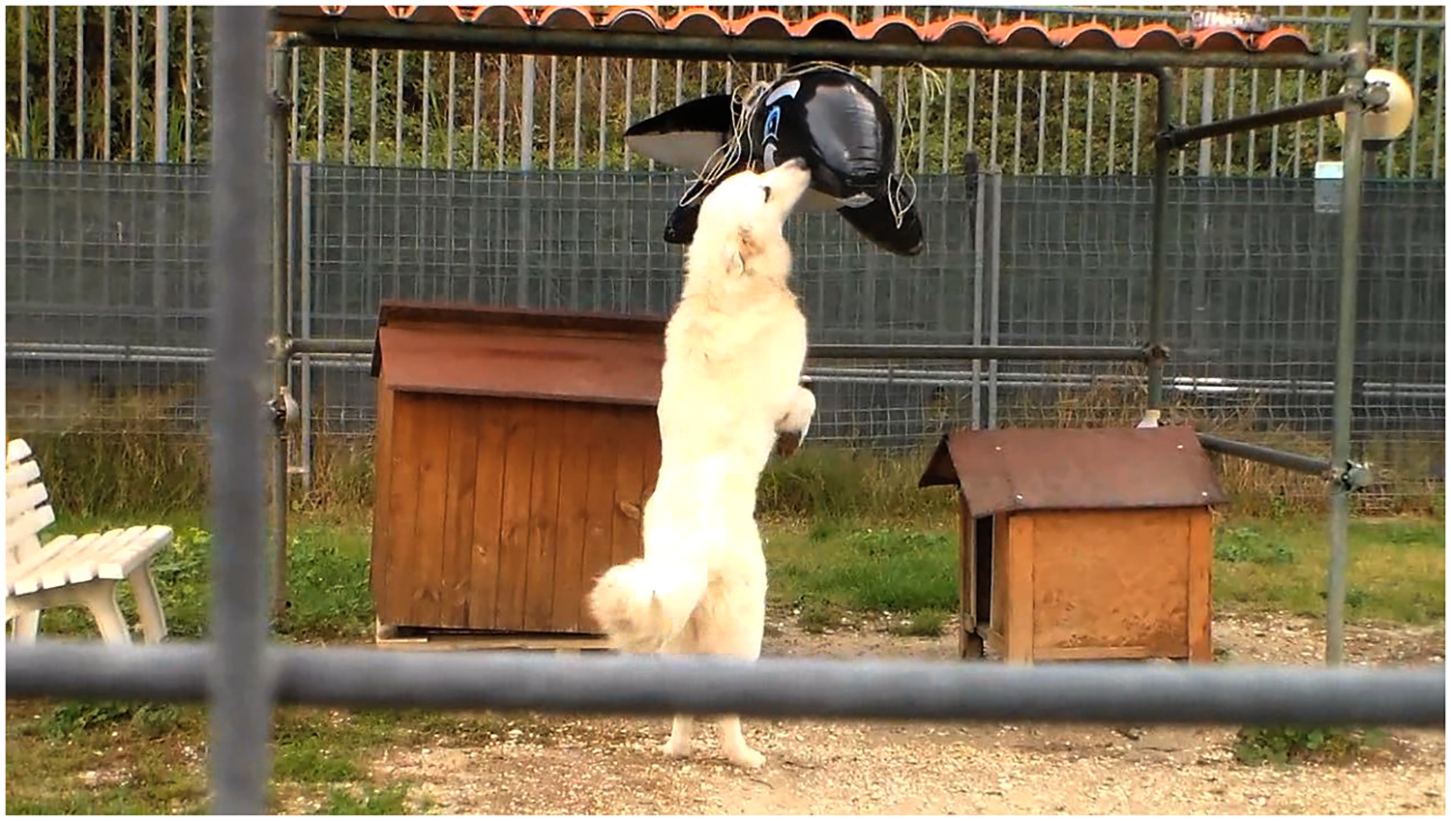
The inflatable killer whale tied to a string under a canopy within the enclosure where the novel object test was performed. The dog was released and was alone in the enclosure. Printed with permission from Simona Borruso original copyright [original copyright 2013].

The enclosure had a canopy where the inflatable object was tied to a string so that it hung just a little bit above the dog’s head. This placement ensured the dog saw the object (Fig 1).

We transformed the dog behavioural approach to the novel object (NO) in a binary variable, where "1" identified a positive approach (sniffing, looking while wagging the tail, inviting to play), and "0" identified a negative approach (barking, growling, threatening, fleeing from) (Table 2). We measured the time latency (min) to approach to the inflatable minus the time latency to looking at the object for the first time; in fact, some dogs were more sensitive to the stimuli furnished by the environment than others, and we wanted to precisely measure the gap between the moment at which the dog had noticed the novel object and the moment at which the animal decided to approach it.

**Table 2:** The ethogram utilised in this study for the observational session in the novel object test.

Behavioural patterns	Operational definition
Sniff the novel object (1)	Putting the muzzle on the object, the dog sniffs the inflatable, investigating it without showing fear.
Look at the novel object while wagging the tail (1)	Looking at the inflatable when near it, or from a moderate distance, the dog wags the tail.
Invite the novel object to play (1)	Bending down with the forelegs outstretched on the ground and the rump upwards, the dog invites the inflatable to play while near it or from a moderate distance.
Bark at the novel object (0)	Looking closely at the inflatable, from a moderate distance, or from a considerable distance, the dog barks at it.
Growl at the novel object (0)	Looking closely at the inflatable, from a moderate distance, or from a considerable distance, the dog growls towards it.
Ears down (0)	Looking closely at the inflatable, from a moderate distance, or from a considerable distance, the dog lowers the ears, presses them on the head, or holds them back.
Tail between the legs (0)	Looking closely at the inflatable, from a moderate distance, or from a considerable distance, the dog holds the tail down or tightly between the hind legs and against the belly.
Avoid (0)	Although the dog has clearly seen the inflatable, ignores it and behaves as if it does not exist, but accurately avoids being close to it.
Walk backwards (0)	Looking closely at the inflatable, from a moderate distance, or from a considerable distance, the dog walks backwards, clearly frightened.
Fleeing from the novel object (0)	Looking closely at the inflatable, from a moderate distance, or from a considerable distance, the dog flees further away from it, clearly frightened.

The reactions of the dogs were classified as positive (1) or negative (0).

### T-maze test

**Fig 2:**
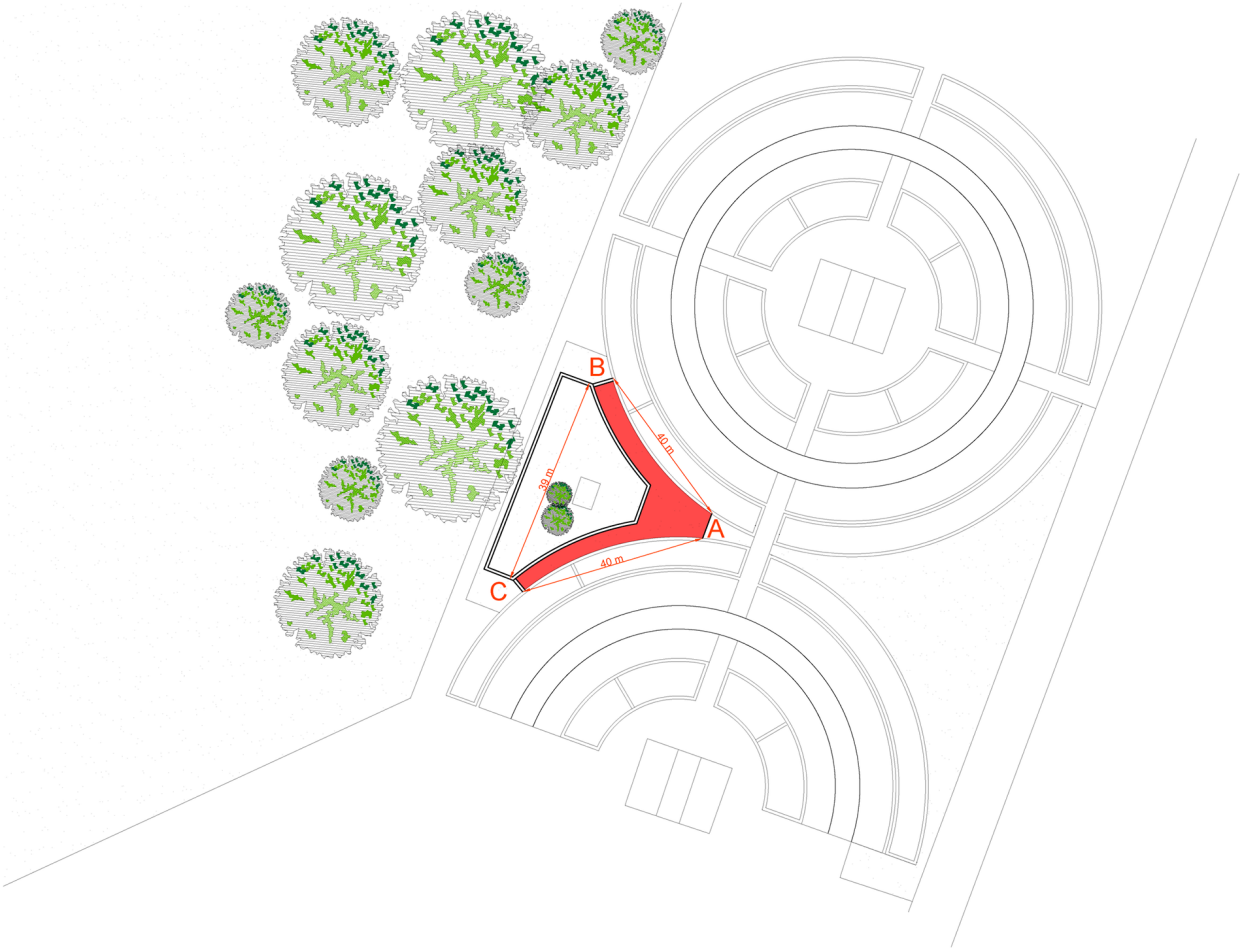
The facility utilised to carry out the T-maze test: A pre-structured enclosure with a "V" shape. Each arm of the structure measures 40 m, and the arms are separated by a distance of 39 m. At the end of each arm there is a reward area where some food can be placed. The dog was released at point A and was alone in the maze.

The facility utilised to carry out the T-maze test was a pre-structured enclosure with a "V" shape (Fig 2).

Each arm was 40 m long and positioned a distance of 39 m from the other. At the end of each arm, there was a reward area where some food, a 2-cm-long piece of sausage, could be placed. Each dog was tested 10 times, and the trials were carried out on different days within, or a few days after, the end of the month of health monitoring. The dog was released at point A and was alone in the maze. For the first 5 repetitions, the food reward was placed at the end of the right arm; from the 6^th^ to the 10^th^ repetitions, the food reward was placed at the end of the left arm. Individuals were tested 10 times. The test lasted 20 min/repetition. In each session, we registered the first direction undertaken by the dog and the time latency (min) it took to find the food.

### Blood sampling and determination of physiological parameters

Blood samples were collected from each individual to determine the complete haemogram, white blood cell differential count, haptoglobin, lysozyme, CD4+/CD8+ ratio, serum proteins (α1-, α2-, β- e γ-globulins), leishmanial and ehrlichia diagnostic tests and oxidative status by the amount of oxidative damage (dRoms), as well as the total antioxidant capacity to cope with it. For each dog, three blood tubes were obtained, one with K3-EDTA and two without K3-EDTA, depending on the type of examination. Concerning the latter, serum was separated by centrifugation and then frozen (−20 °C) until assayed.

#### Haematology

A complete blood cell count (haemogram) was performed using the automated counter Cell-Dyn 3700 (ABBOTT—12 parameters) to assess leukocyte (neutrophils, eosinophils, basophils, monocytes and lymphocytes) counts.

#### Phenotype of lymphocyte subpopulations

Flow cytometric immunophenotyping of leucocytes was performed by analyses of whole blood. The concentration and percentage of CD4 and CD8 T cell populations were measured using a mAb against canine CD4 FITC/CD8 RPE (DC048) purchased from AbD Serotec (Bio-Rad) (UK). Aliquots of 100 μl of whole blood containing approximately 1x10^6^ white blood cells were added to 10 μl of mAbs and incubated for 1 hour in the dark at 4°C. Next, the erythrocytes were lysed, and the leucocytes were fixed by addition of FACSLyse (Becton Dickinson Biosciences, San Josè, CA, USA). After two washes with PBS, the cells were analysed by Cell Quest Pro software on a FACSCalibur flow cytometer (Becton Dickinson Biosciences) equipped with a 488 nm wavelength argon laser and a 635 nm wavelength red diode laser. The results were reported as the percentage of gated cells positive for each cell surface marker.

#### Oxidative stress

The oxidative status was assessed by the amount of oxidative damage (dRoms) as well as the total antioxidant capacity to cope with it. Reactive oxygen metabolites (dRoms) were determined by a colorimetric method. The results of the dRoms test were expressed in arbitrary units called “Carratelli units” (CARR U), that is 1 CARR U = 0.08 mg of H2O2/100 ml (Diacron Adsorbent Test, Grosseto, Italy; limit of detection = 11 CARR; limit of quantification = 40 CARR; linearity 40–1000).

The Total Antioxidant Status test (Randox Lab. UK) was performed by a colorimetric method, and the results are expressed in mmol/l (Diacron Adsorbent Test, Grosseto, Italy; limit of detection = 25 micromol/ml; limit of quantification = 80 micromol/ml; linearity 80–500 micromol/ml).

#### Innate immune response

Lysozyme in canine serum was determined according to established procedures by the bacteriological assay [37–39]. Protein electrophoresis was performed using an automated analyser (Interlab G26).

#### Acute phase protein

Haptoglobin was determined by an immunoenzymatic test (Tridelta Development Ltd. Ireland).

### Statistical analysis

Behavioural patterns utilised to collect data during the observations of dogs when in their cage were grouped into categories (Table 3). The personalities of the dogs were determined by principal component analysis (PCA) utilising the categories of behavioural patterns that were displayed by at least two-thirds of the dogs in the study (see Table 3).

**Table 3:** The behavioural categories utilised for the PCA.

BEHAVIOURAL CATEGORIES	BEHAVIOURAL PATTERNS
**1. Activity**	Standing, walking, trotting, galloping, entering the indoor and going out into the outdoor part of the cage.
**2. Aggressivity towards humans**	Growling, sideways glance, piloerection, curling lip, showing teeth, dashing at bars*.
**3. Anxiety (3.1. plus 3.2.)**	3.1. Body shaking, scratching, muzzle licking, yawning, spinning in place*, autogrooming; 3.2. repetitive pacing in circles, licking or biting bars and object repeatedly, catching “invisible flies”, coprophagy, self-mutilation.
**3.1. Displacement activities**	
**3.2. Stereotyped or repetitive behaviour**	
**4. Attentiveness (4.1. plus 4.2.)**	4.1. Raising ears, looking outside the cage, looking outside the cage carefully, looking at the observer, at unknown people, at the volunteer, at another dog, raising one foreleg, raising both forelegs on the wall or on the bars; 4.2. smelling their environment, smelling the air, smelling the observer, smelling unknown people, smelling the volunteer, smelling another dog.
**4.1. Attention**	
**4.2. Olfactory investigation**	
**5. Dominance towards humans**	Staring at, tail still, stiff body, raised tail, wagging with the tail held high, pricked-up ears (all directed towards humans).
**6. Dominance generic**	Staring at, tail still, stiff body, raised tail, wagging with the tail held high; pricked-up ears (not directed to a specific recipient), urinating with a raised leg.
**7. Subordination towards humans**	Avoiding eye contact, lowering head, flattening ears, cringing, holding the tail down or tightly between the hind legs and against the belly, laying down on the back exposing the ventral side of the chest and sometimes the abdomen, walking backwards (all directed towards humans).
**8. Subordination generic**	Flattening ears, cringing, holding the tail down or tightly between the hind legs and against the belly, laying down on the back exposing the ventral side of the chest and sometimes the abdomen, walking backwards (not directed towards a specific recipient).
**9. Vocal communicability**	Barking, whining, grumbling, mumbling, howling, snorting.
**10. Sociability towards humans**	Wagging the tail, presenting a paw, soliciting to be cuddled, leaning on bars (all directed towards humans).
**11. Sociability generic**	Wagging tail (not directed towards a specific recipient).
**12. Quiet-laziness**	Sitting, laying, dozing.
Playing*	Inviting to play, answering inviting to play, showing object.

*Behavioural patterns excluded from the PCA because they were performed too rarely.

The PCA factors were named based on variables showing correlations with them (significance set at 0,40); furthermore, we utilised the dog’s individual score for each factor to calculate a numerical index for each dog. To obtain that index, the individual scores for each factor were divided by the square root of the eigenvalue of that factor to obtain new scores with homogenous variability. The scores were then ordered in a decreasing sequence for the 28 dogs. The final personality index (PI) was obtained by dividing the individual score for the factor named “Insecurity” by the sum of the individual scores for two factors named “Self-confidence and Sociality twd humans”. A low ratio indicated a bold individual.

We ranked the dogs from the lowest to the highest ratio, i.e., from the boldest to the shyest.

To explain individual variability in vulnerability to diseases, we ran a generalised linear model (logistic regression) using the personality index as a predictor variable, the dichotomous variable “health improvement = 1, worsening = 0”, as a dependent variable and sex, age classes, breed and housing conditions (alone vs. with partner) as factors.

The model considers both simple and interactive effects (saturated model). Given that no relationship was found between the independent variables mentioned and the dependent variables, we ran a stepwise procedure. We checked the linearity of the log ODDs (probability of events and probability of non-events ratio), and the personality index was linear.

To identify the relationship between the personality index and the reaction to the novel object, we again applied a generalised linear model (logistic regression) using the personality index as a predictor variable, the binomial variable “fear towards the novel object” = 0, “non-fear towards the novel object” = 1”, as the dependent variable and sex, age classes, breed and housing conditions as independent factors. Based on a positive and negative approach to the inflatable object, two groups emerged: dogs that did not show any fear, and dogs that were frightened by the novel object. The same model was applied to the two groups, separately. Due to the small sample size of one of the two groups, we did not take into account sex, age classes, breed and housing conditions as factors. The same assumptions were checked.

Furthermore, we applied a linear regression model using the personality index as the predictor variable and the latency to approach the novel object as the dependent variable.

To identify the relationship between the personality index and the latency to find the food in the T-maze test, we applied a linear regression model using the personality index as the predictor variable and the average latency to find the food for each session as the dependent variable. Due to the small sample size, we did not take into account the sex, age classes, breed and housing conditions as factors. Gauss-Markov conditions were verified through the residual analysis.

The difference in mean latency of approaching the inflatable object between the groups was tested by a Mann-Whitney U test because of the different sizes.

The Spearman rank correlation coefficient was applied to assess the relationship among the parameters in the first and, separately, in the second blood sample.

Finally, we also applied a mixed model ANOVA—a between-subjects variable (bold vs. intermediate vs. shy classification) and a within-subjects variable (different parameters at time 1 vs. time 2).

All statistical analyses were carried out using Statistica release 8 (StatSoft Inc., Tulsa, OK, U.S.A.).

### Ethics statements

We did not need an institutional or governmental permission to carry on the study since it was an observational study that involved two blood samples, the first enforced by the Italian National Law on dogs entering a shelter, and the second falling in good veterinary practice allowed by the National and International Laws.

Neither anesthesia nor euthanasia, or any kind of animal sacrifice, was part of the study.

## Results

### Dog personality assessment

The first three factors in the PCA together explained approximately 56,31% of the total variance of the data. For the first factor, any correlation of 0.50 or above was deemed relevant for the variable loading (Table 4). The first factor (F1) was defined “Self-confidence” since it was highly correlated with measures of “dominance, physical activity, attention, vocalisation, generic sociability"; for the second factor (F2), since it was the only one related to anxiety, we considered relevant any correlation of 0.40 or above. It was defined as "Insecurity” since it was highly correlated with measures of anxiety and subordination towards humans"; the third factor (F3) was defined “Sociality towards humans” since it was highly correlated with measures of sociability towards humans" (see Table 4). Thus, dogs with high F1 and F3 values were individuals with a bold personality because they were active, confident and sociable; in other words, they showed some degree of confidence in themselves. In contrast, dogs with high F2 values were individuals with a shy personality because they showed a high frequency of displacement activities and stereotypical behaviour, as well as subordinate attitudes towards humans; in other words, they were insecure.

**Table 4:** Results from the PCA.

BEHAVIOURAL CATEGORIES	Factor 1	Factor 2	Factor 3
	Self confidence	Insecurity	Sociality towards humans
1. Activity	**-0.609412**	-0.187274	0.181743
2. Aggressivity towards humans	0.312369	-0.303038	**0.494560**
3. Anxiety	-0.329097	**-0.432679**	-0.134744
4. Attentiveness	**-0.765243**	-0.426778	-0.067065
5. Dominance towards humans	-0.241730	-0.218453	**0.726856**
6. Dominance generic	**-0.768867**	0.267695	0.327673
7. Subordinate towards humans	0.133386	**-0.578569**	-0.085395
8. Subordinate generic	-0.194147	**0.589041**	0.023476
9. Vocal communication	**-0.600929**	-0.482599	0.056381
10. Sociable towards humans	-0.206619	-0.463207	**-0.696310**
11. Sociable generic	**-0.583830**	**0.528022**	-0.406366
12. Quiet-laziness	**0.629284**	-0.351733	-0.068403

Loadings ≥ 0.40 are shown in bold.

The individual personality index, calculated as described in the Methods section and considering the ‘weight’ of each factor, provided a gradient along which each dog was classified from ‘bold’ to ‘shy‘.

We were able to identify 3 different categories: bold dogs (n = 9), intermediate dogs (n = 10), shy dogs (n = 9). The categories were formed by the observation of the qualitative behaviour of each individual dog matched with the personality index. The first category consisted of dogs that had an index between 0 and 0.3, and the observed behaviour confirmed that class. The second category comprised dogs that had an index value of 0.3 to 0.9, but observation of the qualitative behaviour of two subjects (Pedro and Bistecca) with 0.68 and 0.7 indexes led us to classify them in the third group with index values from 0.9 to 2.25. In fact, Pedro and Bistecca corresponded more to a shy personality description: Pedro was terrified by people and by other dogs; Bistecca was also subordinate to humans and showed fear aggression towards other dogs; this was highlighted, for example, by barking and growling at other dogs whilst holding the tail tucked between the legs.

### Novel object test

Many different kinds of reactions to the novel object were recorded: some dogs were clearly frightened (9 individuals), and some others (19 individuals) were curious and willing to investigate the “strange” object. Thus, once the novel object was noticed, the dogs that showed fear clearly avoided it, ran around in the enclosure with ears and tail held low, tried to maintain a large distance from the inflatable object and barked at it. In contrast, dogs that showed curiosity approached the inflatable object, sniffed it with a relaxed body posture and one dog invited it to play.

**Fig 3:**
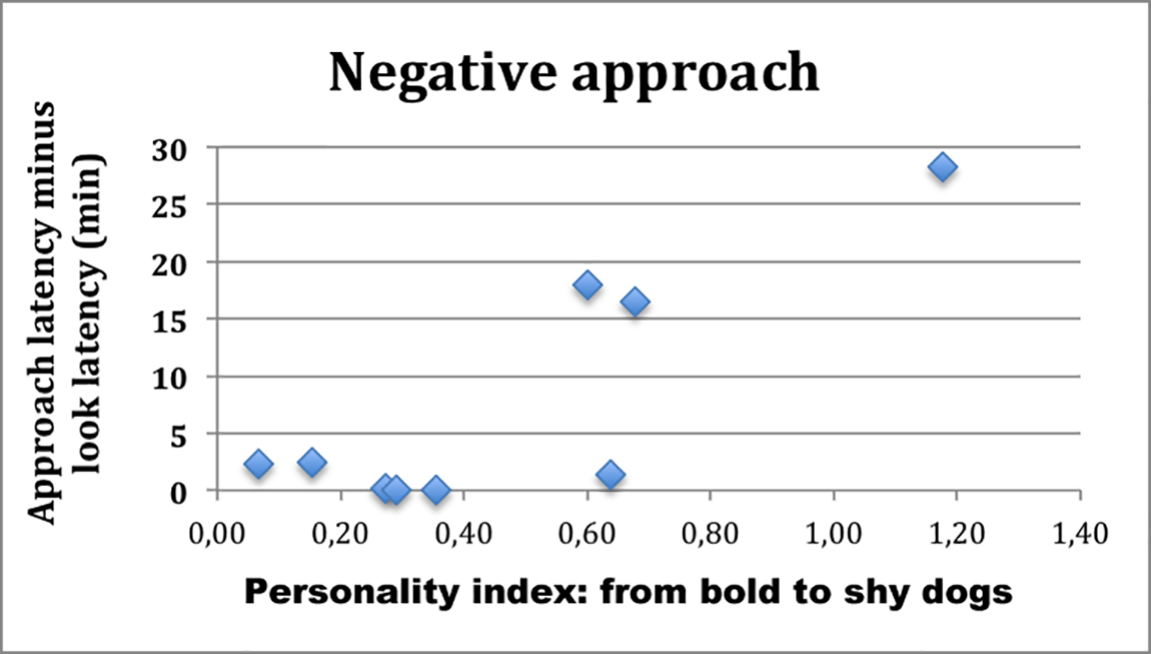
The time latency of approach to the novel object of dogs who showed fear towards the unknown object. Dogs are ordered from left to right from boldest to shyest.

**Fig 4:**
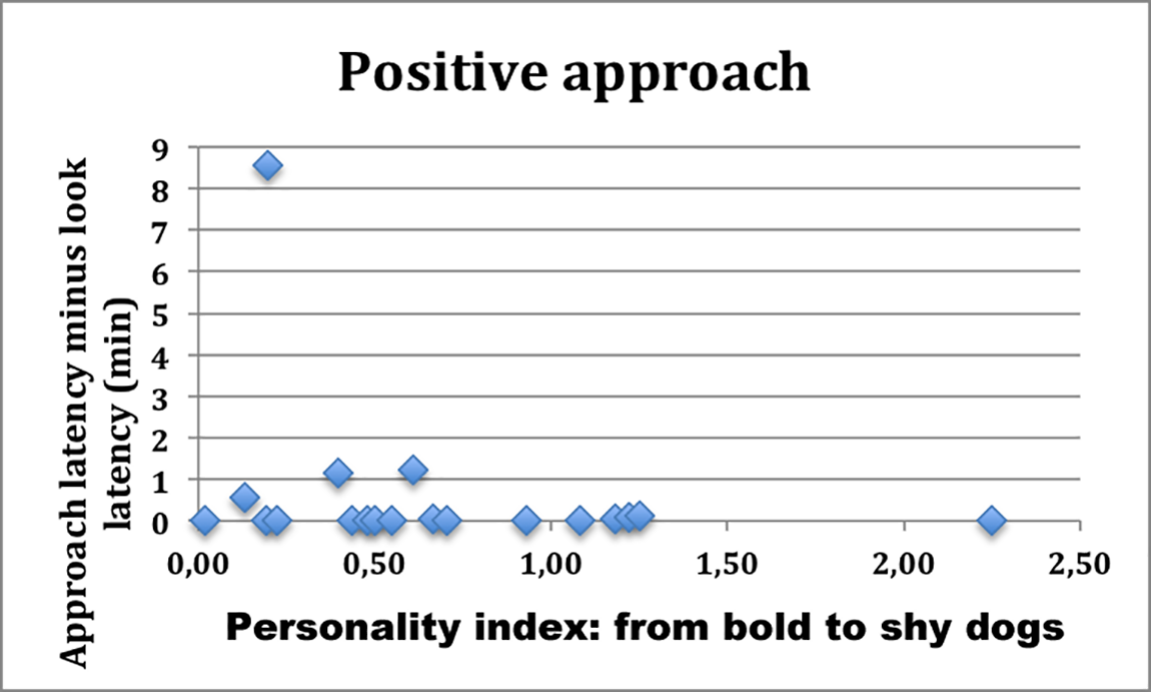
The time latency of approach to the novel object of dogs who did not show fear towards the unknown object. Dogs are ordered from left to right from boldest to shyest.

The personality index and other factors (sex, age class, breed and condition of housing) were not good predictors of the quality (positive or negative) and latency to approach the novel object because, indeed, a number of “bold” dogs showed fear towards the inflatable killer whale (odds ratio = 8,17, N = 28, power = 0,74, p < 0,05) and were not quick to approach and investigate it. Nevertheless, if we distinguished between dogs that had a negative approach and those that had a positive approach, personality seemed to play a role. The two groups differed in mean latency to approach (dogs without fear: 40,53 sec: fearful dogs: 470,67 sec; test U Mann-Whitney: Z = -2,78; Z_agg_ = -2,79; p = 0,005). Among the dogs that showed fear, bolder dogs were clearly faster (bold = 1,23 min; intermediate = 6,48 min; shy = 22,40 min) (r^2^ = 0,72; df = 1,7; p = 0,0041) (Fig 3); however, among the dogs that did not show any fear, the behaviour of bold and shy dogs did not differ (r^2^ = 0,07; df = 1,17; p = 0,29) (Fig 4).

### T-maze test

**Fig 5:**
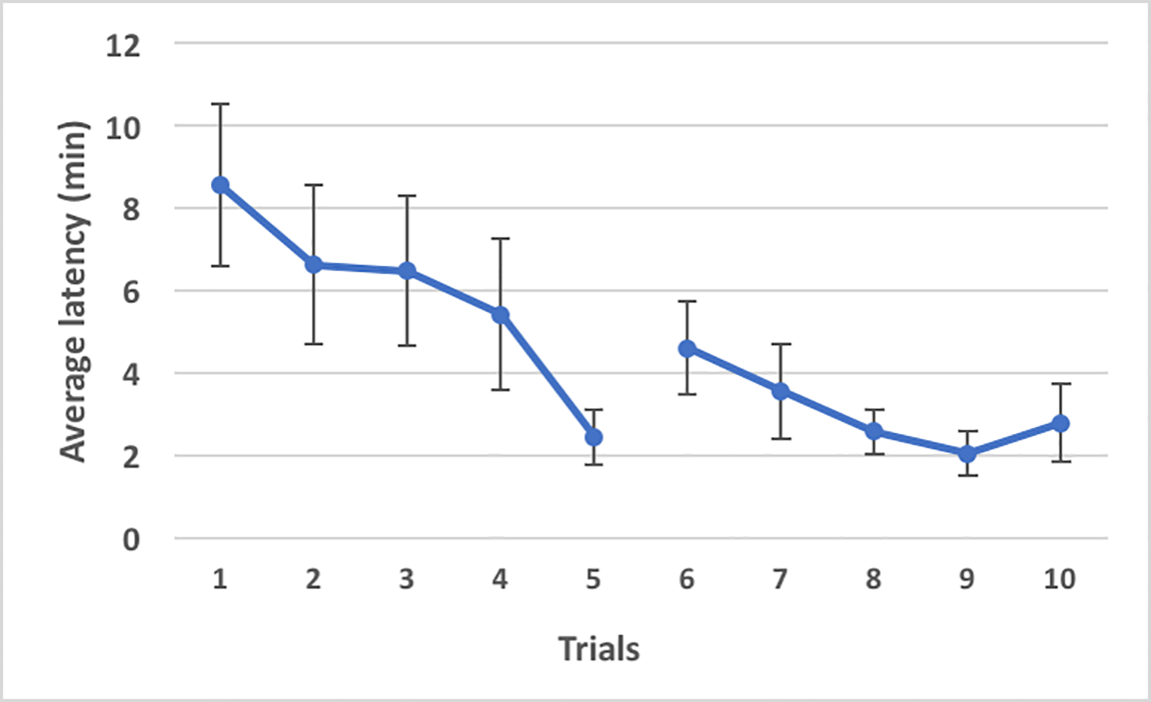
The average time latency (minutes) that the 28 dogs spent searching food in the T-maze test in the 10 trials. In the 6^th^ trial, the location of the food was changed from the right to the left arm. Bars indicate the standard error.

The latency to find food tended to decrease from the 1^st^ to the 10^th^ trial (Fig 5). In the first five trials, when food was placed at the end of the right arm of the maze, the average of all dogs together ranged from 8,54 (1^st^) to 2,44 (5^th^) minutes.

In the 6^th^ trial, when the position of the food was moved to the left arm for the first time, the mean time latency increased to 4,60 min and again dramatically decreased during the 9^th^ and 10^th^ trials (2,04 and 2,78 min, respectively) (see Fig 5).

The personality index was not a good predictor of the latency to find the food (r2 = 0,028, p = 0,398).

The first 9 dogs, belonging to the “bold” category, from the 7^th^ to the 10^th^ trials showed the highest mean latency to find the food when compared to intermediate and shy categories of dogs (3,44, 2,22 and 2,59 min, respectively).

**Fig 6:**
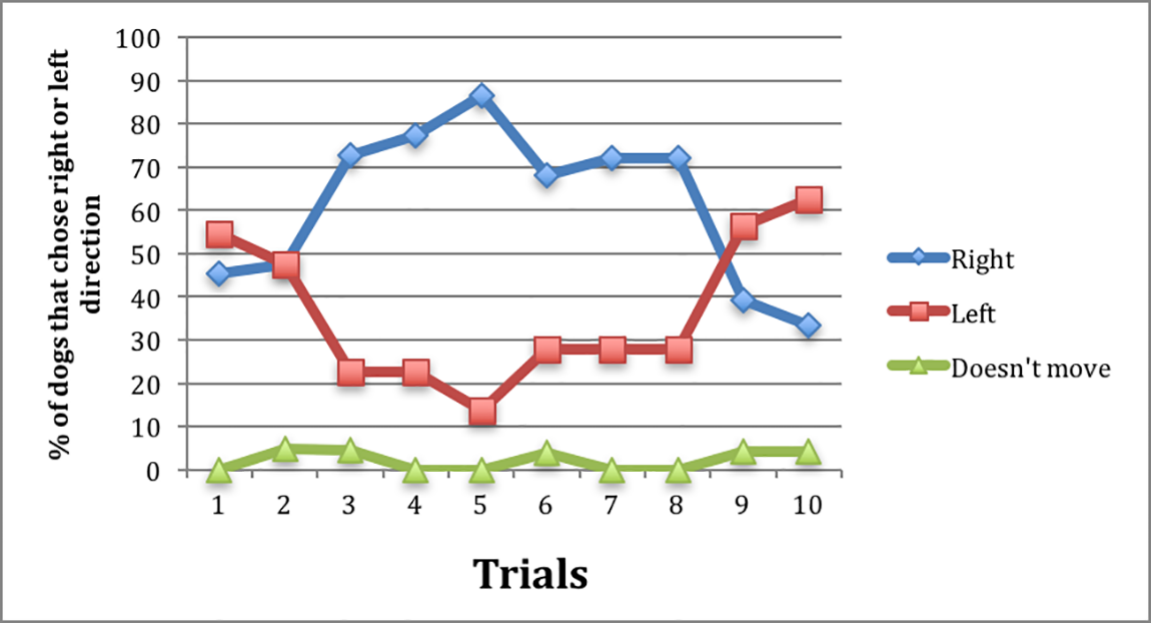
The direction chosen by the 28 dogs in the 10 trials during the T-maze test.

Although the latency to find the food decreased dramatically from 8,54 min (mean of the first trial) to 2,78 min (mean of the tenth trial), the choice of direction taken by the dogs seemed totally random (Fig 6). In other words, neither bold nor shy dogs learned to go in the right direction to find the food; in contrast to findings in other mammal species, in which bold animals were usually slower to learn because they were less prone to routine changes during the reversal learning task, we could not find any relationship between the direction chosen in the T-maze test and dog personality.

### Dog personality and health status

The generalised linear model (logistic regression) developed for the dependent variable ‘health improvement/worsening’ significantly showed that dog personality index was a good predictor of health trend (p = 0,019; personality index odds ratio = 9,72), especially for crossbred vs. purebred dogs (Table 5).

**Table 5:** Modelled probability of the dog health status trend as personality index increases.

Effects of the model	Level of effects of the model	Estimate of the parameters	Standard Error	Wald Stat.	Lower CL 95%	Upper CL 95%	P-value
Intercept		2.33878	0.75946	10.37931	0.91595	3.761604	0.001274
Personality index	Two levels: worsened (0) and improved (1)	-2.33303	0.992329	5.52750	-4.27796	-0.388099	0.018720
Breed	Two levels: P (1) and M (0)	0.07985	0.363698	0.04820	-0.63299	0.792680	0.826230
Scale		1.00000	0.00000		1.00000	1.000000	

$P\left(H=1\right)=\frac{\text{exp}\left\{0.7595-2.33\left(PI\right)+0.0798(PB)\right\}}{1+\text{exp}\left\{0.7595-2.33\left(PI\right)+0.0798(PB)\right\}}$

PI = Personality index (continuous variable).

PB = Pure breed (discrete variable PB = 1; MB = 0).

H = Health status (dichotomous variable H = 0 worsened; H = 1 improved).

When the personality index (PI) increases and PB is equal to MB, P(H) increases.

**Fig 7:**
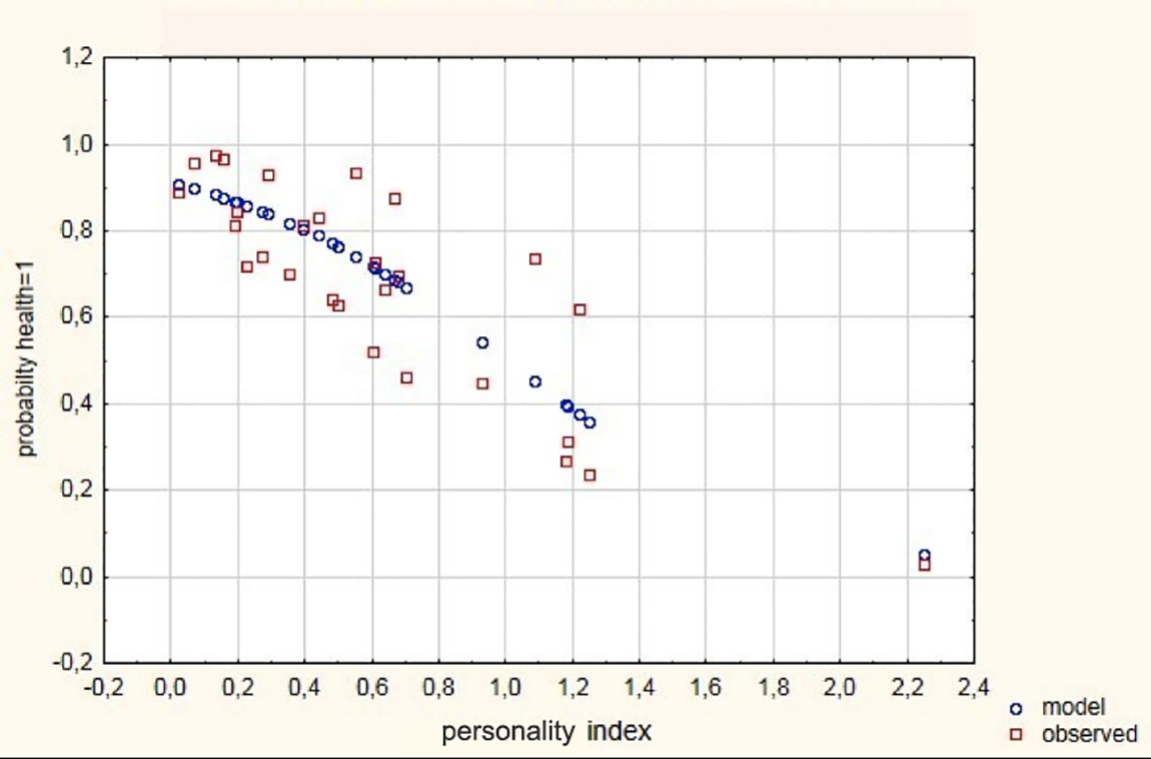
Scatterplot of observed and expected probability against the personality index. Health improvement = 1, worsening = 0.

The model showed a good fit to the data (Fig 7).

Thus, the bolder the dogs were, the lower was the probability of contracting a disease and/or the higher was the probability of improving their health status, especially for crossbred dogs. The results shown in Table 5 confirm that as the personality index (PI) increased towards higher shyness trait scores, the probability of an improvement in health status decreased (logistic regression coefficient for personality index = -2,33).

These findings indicated that the symptoms detected during the 30 days of intensive monitoring of the 28 dogs’ health were confirmed by physiological parameters (Table 6).

**Table 6:** Dog health status trend during the 30 days of accurate monitoring.

Dog name (ordered from boldest to shiest)	Personality Index (low value = bold, see par. 2.7.)	Breed	Conditions at entry into the shelter	Symptoms developed within 30 days of monitoring	Trend of dog health status during the 30 days of accurate monitoring
Ettore	0.02	M	Good	None	Improved
Bravery	0.07	P	Good	None	Improved
Margot	0.13	M	Good	None	Improved
Peggy	0.15	P	Good	None	Improved
Benjo	0.19	P	Good	None	Improved
Pippo**	0.2	M	Good	Inflammation of the penis	Worsened
Bho	0.22	P	Good	None	Improved
Ribes	0.27	M	Good	None	Improved
Rott	0.29	P	Good	None	Improved
Pinza*	0.35	M	Good	Cough reverse and bloody diarrhoea	Improved
Ercole	0.39	P	Good	None	Improved
Sofficino Gandhi	0.44	M	Good	None	Improved
Ululì**	0.48	M	Dislocated hip-joint	Diarrhoea	Worsened
Bugs Bunny	0.5	M	Good	None	Improved
Ab	0.55	M	Good	None	Improved
Frollo**	0.6	P	Good	Diarrhoea	Worsened
Vecchio Rex**	0.61	P	Good	Bilateral otitis	Worsened
Pis	0.64	M	Poor conditions. parasitic otitis and ectoparasites	None	Improved
Mary*	0.67	M	Good	Diarrhoea	Improved
Pedro*	0.68	P	Good	Diarrhoea	Improved
Bistecca**	0.7	P	Good	None	Worsened
Max	0.93	P	Good	None	Improved
Chica**	1.08	M	Good	Diarrhoea	Worsened
Pedalino**	1.18	M	Good	Diarrhoea	Worsened
Ugo**	1.18	P	Good	Vomiting. diarrhoea and loss of appetite	Worsened
Sally	1.22	P	Good	None	Improved
Nervo	1.25	M	Good	None	Improved
Schizzo**	2.25	M	Good	Vomiting and diarrhoea	Worsened

The 28 dogs are listed from boldest to shyest.

* = dogs that showed some symptoms (e.g., diarrhoea, cough, urinary infection, among others);

** = dogs that, other than showing some symptoms, showed an increase in physiological parameters considered markers of inflammation.

M = mongrel; P = purebreed.

**Fig 8:**
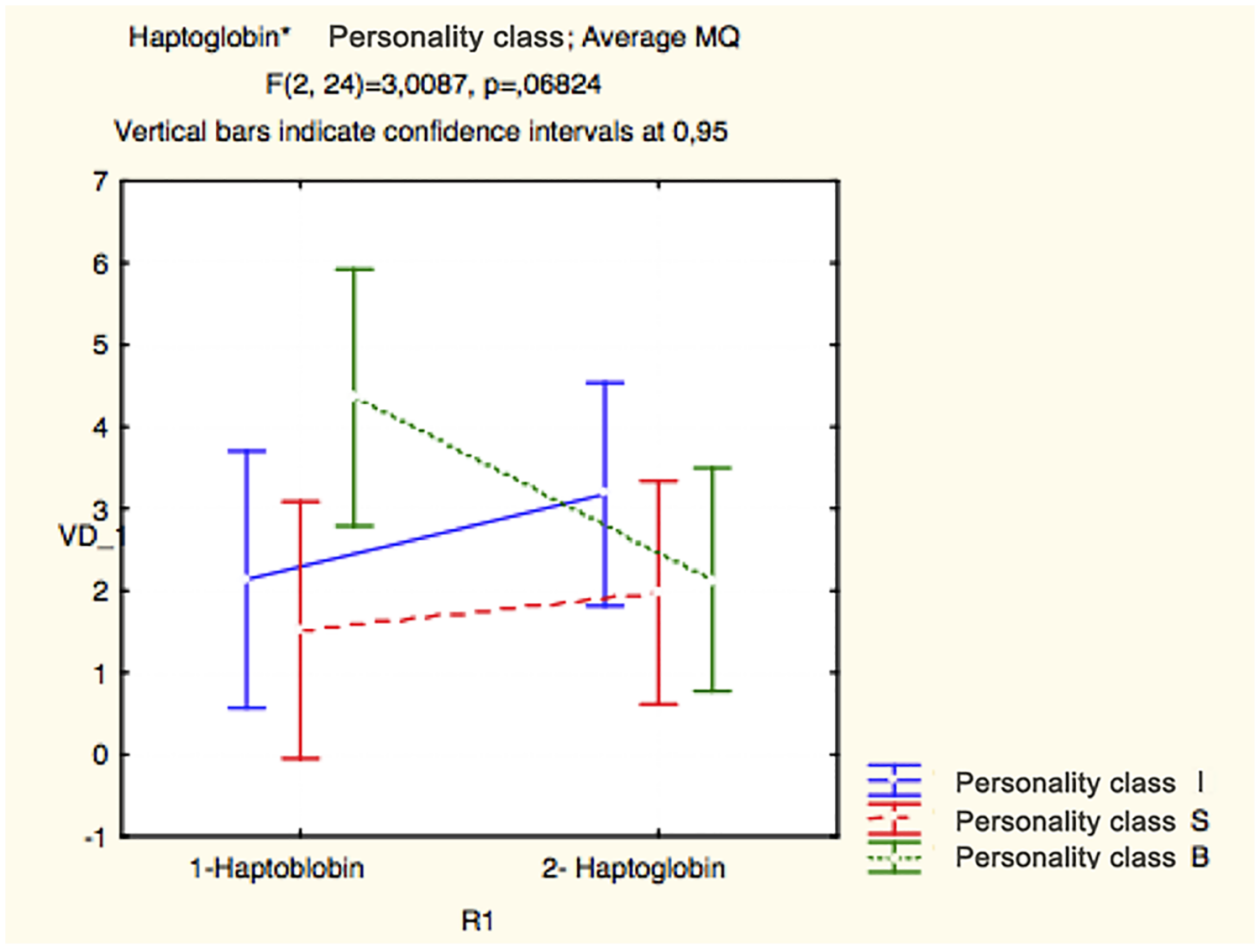
Mixed model ANOVA—A between-subjects variable. The results of the mixed model ANOVA—a between-subjects variable (bold vs. intermediate vs. shy personality class) and a within-subjects variable (haptoglobin values of the 1^st^ blood vs. the 2^nd^ blood sample).

**Fig 9:**
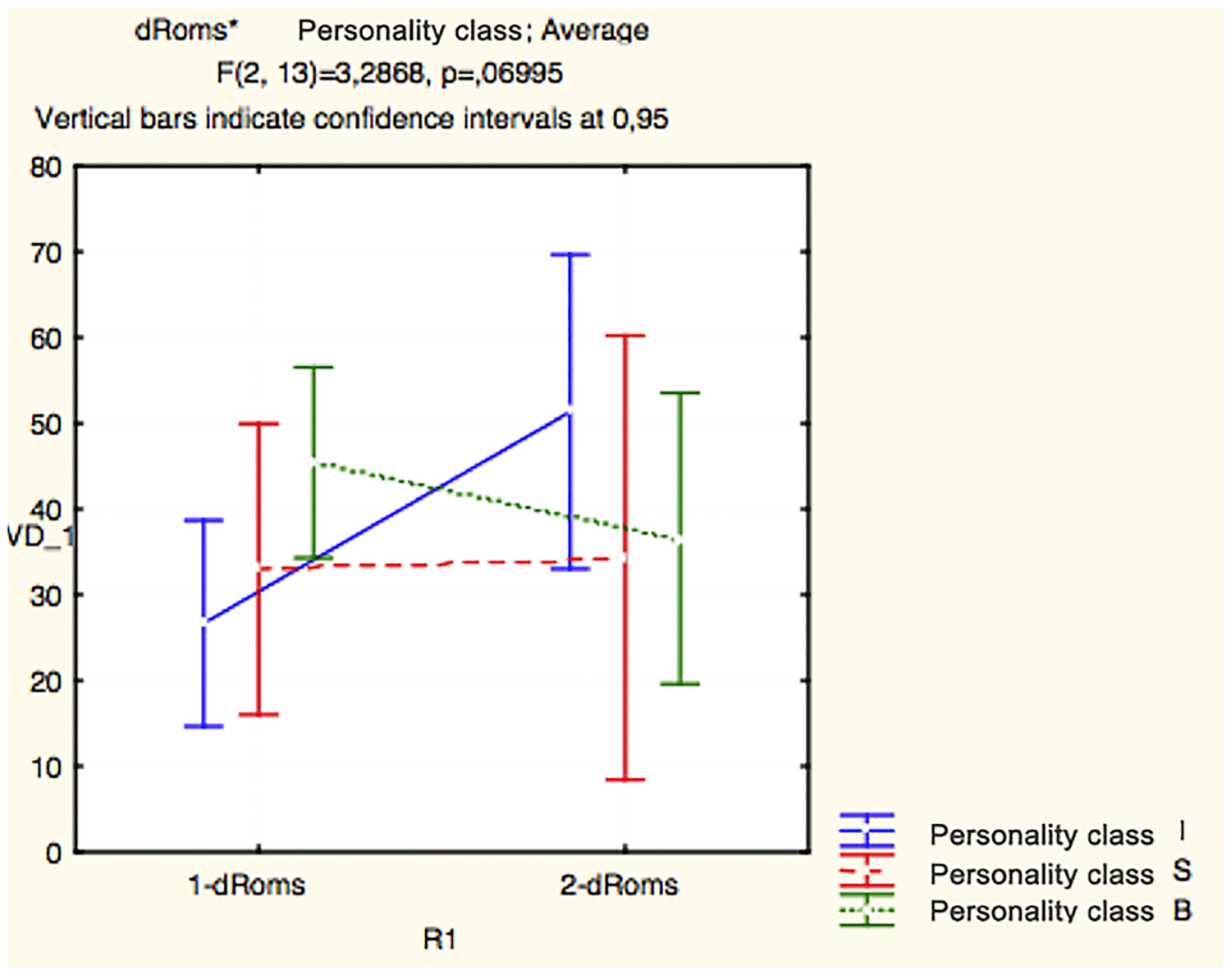
Mixed model ANOVA—A within-subjects variable. The results of the mixed model ANOVA—a between-subjects variable (bold vs. intermediate vs. shy personality class) and a within-subjects variable (dRoms values of the 1^st^ blood vs. the 2^nd^ blood sample).

The physiological evidence that bolder individuals reacted better to the stressful shelter environment was confirmed by comparison of specific parameter values in the first and second blood samples. For example, bolder dogs had the highest mean level of haptoglobin in the first blood sample (B = 3,71 mg/ml; I = 2,05 mg/ml; S = 1,52 mg/ml), but it decreased dramatically after 30 days, compared to the other classes of dog personality (B = 2,12 mg/ml; I = 3,18 mg/ml; S = 1,98 mg/ml), and this difference tended to be significant (ANOVA for repeated measures: personality*haptoglobin: F_2,24_ = 3,001; p = 0,06) (Fig 8). The same was true for the level of dRoms (ANOVA for repeated measures: personality*dRoms: F_2,13_ = 3,29; p = 0,06) (Fig 9), despite the small number of dogs, further reducing the number of samples due to technical problems during the blood analysis (Table 7).

**Table 7:** Blood parameter average values of blood samples in the different personality classes.

First blood sample	Bold	Intermediate	Shy
**Leukocytes**	14,91	13,84	15,14
**Neutrophils**	10,21	10,71	11,56
**Lymphocytes**	1,94	1,26	1,56
**Monocytes**	1,37	1,20	1,37
**Eosinophils**	1,21	0,55	0,53
**Red cells**	7,07	7,36	7,22
**Haemoglobin**	17,36	17,52	17,22
**Haematocrit**	51,26	51,76	51,19
**Lysozyme (μg/ml)**	3,76	4,89	5,18
**Serum protein**	6,94	6,85	6,90
**Albumin/Globulin**	0,80	0,85	0,80
**Haptoglobin**	3,71	2,05	1,52
**CD4/CD8**	3,08	2,28	2,57
**Total antioxidants**	401,44	438,75	434,67
**dRoms**	40,88	33,13	42,33
**Second blood sample**			
**Leukocytes**	12,66	12,24	14,14
**Neutrophils**	8,48	8,33	9,39
**Lymphocytes**	1,81	1,53	2,36
**Monocytes**	0,86	1,29	1,33
**Eosinophils**	1,38	0,95	0,96
**Red cells**	7,60	7,24	7,53
**Haemoglobin**	18,43	17,17	17,51
**Haematocrit**	53,94	50,52	51,89
**Lysozyme (μg/ml)**	2,61	4,45	5,02
**Serum protein**	6,63	6,72	6,57
**Albumin/Globulin**	0,85	0,77	0,78
**Haptoglobin**	2,12	3,18	1,98
**CD4/CD8**	2,89	1,95	2,57
**Total antioxidants**	345,33	277,50	306,14
**dRoms**	33,25	48,86	26,17

Finally, several blood parameters correlated amongst each other in the first blood sample (Table 8), and their number increased after 30 days, i.e., in the second blood sample (Table 9). It is interesting to note that some correlations between blood parameters were moderate for all 28 dogs, but increased in significance for bold dogs and decreased for intermediate and shy dogs, when personality categories were considered separately (e.g., albumin/globulin ratio and haptoglobin. All dogs: rho = - 0,41, N. = 28, p < 0,05; bold dogs: rho = - 0,70, N. = 9, p < 0,05; intermediate dogs: rho = - 0,08, N. = 10, NS; shy dogs: rho = - 0,53, N. = 9, NS).

**Table 8:** Significant correlations (p < 0.05) among blood parameters in the first blood sample (Spearman rank correlation test).

Blood parameters	Rho
Leukocytes—Neutrophils	0.91
Leukocytes—Monocytes	0.58
Neutrophils—Monocytes	0.50
Lymphocytes—Eosinophils	0.60
Red cells—Haemoglobin	0.88
Red cells—Haematocrit	0.91
Haemoglobin—Haematocrit	0.95
Neutrophils—Eosinophils	- 0.41
Eosinophils—Lysozyme	- 0.40
Lysozyme—CD4/CD8 ratio	- 0.42

**Table 9:** Significant correlations (p < 0.05) among blood parameters in the second blood sample (Spearman rank correlation test). *Indicates correlations that were not present in the first blood sample.

Blood parameters	Rho
Leukocytes—Neutrophils	0.93
Leukocytes—Monocytes	0.65
Neutrophils—Monocytes	0.68
Lymphocytes—Eosinophils	0.47
Lymphocytes—Red cells	0.40 *
Red cells—Haemoglobin	0.77
Red cells—Haematocrit	0.80
Haemoglobin—Haematocrit	0.95
Haematocrit—Albumin/Globulin ratio	0.39 *
Albumin/Globulin ratio—CD4/CD8 ratio	0.47 *
Lymphocytes—dRoms	- 0.48 *
Monocytes—Eosinophils	- 0.45 *
Monocytes—Total antioxidants	- 0.49 *
Eosinophils—Lysozyme	- 0.39
Red cells—Lysozyme	- 0.46 *
Haemoglobin—Lysozyme	- 0.56 *
Haematocrit—Lysozyme	- 0.50 *
Lysozyme—CD4/CD8 ratio	- 0.39
Serum protein—Albumin/Globulin ratio	- 0.43 *
Serum protein—CD4/CD8 ratio	- 0.57 *
Albumin/Globulin ratio—Haptoglobin	- 0.41 *

## Discussion

### Dog personality and health status

Direct observation of dog behaviour using traditional ethological methods [36] yielded a dataset that, analysed by means of PCA, identified three personality dimensions: "self-confidence”; "Insecurity” and "sociality towards humans”. None of the three identified dimensions alone was sufficient to assess the personality of the dogs in this study along the proactive and reactive axis since a bold individual is much more than an individual who is aggressive, dominant or fast in taking decisions; on the other hand, a shy individual is much more than an individual who is quiet, subordinate or slow in taking decisions [31]. For example, in contrast to potential expectations, researchers observed that bold individuals of some species are less able to solve problems caused by a routine change, whereas shy individuals can adapt more quickly to changes [1, 40–41]. For these reasons, we considered the variables that featured high on the first factor as important variables that featured high on the third factor in relation to boldness, and, conversely, of the variables that featured high on the second factor; thus, we wanted to create a (weighted) index that combined the three factors, providing a broader personality dimension. Such an index allowed us to assess dog personality along the boldness-shyness axis, in accordance with the literature (see for example [29, 35, 42–45]).

In fact, most of the literature on the subject extract primary factors that are subsequently combined in a broader dimension along the bold-shy continuum. For example, Svartberg and Forkman ([29] p. 152) state the following: <<The higher-order factor, which relates to playfulness, exploration, interest in chase and sociability, indicates that to a great extent it is one single dimension that influences the dogs’ behaviour during the whole test>> and (p. 153) <<Shy individuals are generally cautious, timid and evasive in novel situations—both in social and in non-social situations—while bolder individuals are more spontaneous, social, and exploratory>>. Moreover, Svartberg ([30] p. 16) note the following: <<I calculated a boldness score, by averaging the scores for playfulness, curiosity/fearlessness and sociability.

Furthermore, the results presented herein suggest that dogs rated higher in ‘boldness’ are more likely to possess the skills to cope with a stressful environment, which potentially carries a high risk of infection, such as a dog shelter. This finding is in accordance with previous findings reported for other mammalian species (for mice see, for example, [1, 17, 46–47]). In other words, the results of this study suggest that there is a relationship between personality and stress-related disease vulnerability. In fact, the health of bold dogs even improved after a stay of one month in the shelter, while intermediate or shy dogs tended to worsen because the latter have an inadequate stress response, due to a scant capacity to control the environment. It has been suggested ([1, 17, 46–50]; [51] reviewed in [52]) that physiological and behavioural changes occur when an organism is not able to control the environment, leading to an increased vulnerability to psychosomatic diseases, e.g. cardiovascular, gastrointestinal, and immunological disturbances [52]. Thus, since the hypothalamic-pituitary-adrenal axis is an important mediator in communication between the brain and the immune system [24–25, 53–56], lowering of the immune system defences in stressed shy individuals leads to an increased vulnerability to diseases.

Furthermore, the present results confirm that one personality dimension that is particularly important for determining disease vulnerability seems to be ‘sociability’, in agreement with the study by Capitanio et al. [57] on rhesus macaques. Sociability in dogs housed in a shelter has been found to be related to a higher total antioxidant capacity [58] and a lower concentration of faecal cortisol metabolites [35], both of which are potentially associated with higher levels of welfare.

An added value of the current results is that dogs were tested in a "natural setting" (referring to the infective agents), such that the observed dogs were presented with a stressful situation replete with infectious agents that pre-existed in the dog shelter, rather than artificially inoculating them with infective agents. Especially in Italy, where the “no kill policy” has been enforced by law since 1991 (and since 1988 in the Lazio Region) (see [58], p. 228), shelter dog populations might be an ideal subject for studying the effects of ‘naturally occurring stress’ on physiology and immunology. One point of the same law states the following: <<Shelter dogs cannot be used as experimental animals>> (National Italian Law No. 281 come out in 1991). Thus, we must stress that we are not suggesting the use of shelter dogs as experimental subjects, but from a perspective of animal welfare, we suggest that the physiological status of dogs housed in shelters should be monitored closely to study their reaction to stress, as an alternative to officially authorized laboratory experiments on dogs with the same purpose.

As suggested by Capitanio et al. [57] for adult male rhesus monkeys, there are physiological correlates of personality factors, and the relationship between personality dimensions and physiological measures reflect the particular demands of the situation experienced by the animals. The blood parameter values found in this study support this perspective. First, it is well known that in dogs, as well as in other species, general leucocytosis, neutrophilia, lymphopenia, and eosinopenia occur during acute stress [25, 53, 59–60], as well as increases in serum and urine lysozyme levels during infections [37, 39]. Leukocyte, neutrophil, lymphocyte and eosinophil numbers, as well as the lysozyme value and CD4/CD8 ratio, displayed substantial temporal consistency in the present study.

Furthermore, some correlations between blood parameters that appeared in the second blood sample were not present in the first one. In some respects, the 30 days of permanence in the shelter provided a clearer picture of the dog’s health.

For example, it is interesting to note the negative correlation between lymphocytes and dRoms (as indicators of free radicals): it is well known that stress provokes decreases in lymphocytes [25] and increases in dRoms [61]. Additionally, the positive correlation in the second blood sample between the albumin/globulin ratio and the CD4/CD8 ratio indicated that both defence systems (humoral and cell-mediated) were engaged because of an infection (predominantly viral).

Still, haptoglobin, an acute phase protein, is correlated to the degree of severity of an infection and is considered a predictive marker of inflammation because it indicates inflammation earlier than the immune responses and the appearance of clinical symptoms [62]. In this study, bolder dogs tended to have higher levels of haptoglobin in the first blood sample, whereas this difference disappeared in the second blood sample; still, there was a negative correlation between the albumin/globulin ratio and the haptoglobin in the second blood sample, especially when personality categories were considered separately.

These observations would further support the idea that the dogs were reacting to infectious agents encountered during the first 30 days of their stay at the shelter and, furthermore, that bold dogs coped with this insult better than shy ones.

Thus, several pieces of evidence showed that the highly infectious environment had an impact on the dogs. Nevertheless, the health of some dogs improved, the majority of which were bold.

The only exception was Pippo, who despite being included in the ‘bold personality’ category, showed a deterioration in his health condition with obvious symptoms of urinary infection emerging during the 30 days of observation.

### Novel object test

Wilson et al. [63] suggested that boldness can be defined as an individual’s general tendency to approach novel objects and willingness to take risks, and indeed, this measure has been used in a number of species (e.g., [64–66]). However, in contrast to such studies, the current results from the Novel Object test did not seem to provide useful information about the personalities of the dogs because bold and shy dogs did not react differently to the inflatable killer whale. King et al. [67] showed in their study that, while not specifically measured, dogs that exhibited postural signs of fear often showed increased latency times to the novel object approach, based on anecdotal observations. Our results, in which the latency time of approach was measured, confirmed this observation but showed that, among dogs that showed fear, personality seemed to again play a crucial role because bolder dogs, although clearly frightened, clearly were faster to approach the unknown object.

### T-maze test

It has been reported for many animal species that bold animals are usually less thorough explorers and rely more on routines [9, 41, 64, 68–69]. In the present study, bold dogs were slower to find the food than intermediate and shy ones, but the difference was not statistically significant. Overall, the performance of dogs in the maze improved over time since the latency to obtain food decreased across trials, but the dogs never learned the correct the rule regarding the direction to take to find the food more quickly, either during the acquisition or during the reversal phase. Thus, it would seem that dogs learned that there was food somewhere, and hence, their speed of movement in the maze increased, but they did not acquire the knowledge of the location in which to find the food.

## Conclusions

In the present study, dogs were monitored for thirty days after their entry into the shelter. The observed increase in leukocytes, neutrophils and eosinophils, as well as the increase and/or decrease in other blood immunological parameters, revealed an immediate reaction to stress and to exposure to diseases present in the shelter that, depending on the personality of the dog, led to the improving or worsening health status.

## Supporting information

S1 Table Raw data regarding behavioural patterns in cages, physiological parameters and reaction of dogs during the the novel object test. (PDF)

S2 Table Raw data regarding latency for finding the food during the the T-maze test. (PDF)
