# Correction: Bold personality makes domestic dogs entering a shelter less vulnerable to diseases

**DOI:** 10.1371/journal.pone.0203399

**Published:** 2018-08-29

**Authors:** The PLOS ONE Staff

## Notice of republication

Incorrect versions of Fig 1 and Fig 2 were published in error. The publisher apologizes for these errors. This article was republished on August 15, 2018 to correct for these errors. Please download this article again to view the correct version.
