# Supplementary material for: Bold personality makes domestic dogs entering a shelter less vulnerable to diseases
Source: PLoS One. 2018 Mar 29;13(3):e0193794. doi: 10.1371/journal.pone.0193794 (PMC5875777; doi:10.1371/journal.pone.0193794)
Supplement: S1 Table — (PDF) [file pone.0193794.s001.pdf]

S1. Table with raw data regarding behavioural patterns in cages, physiological parameters a

NAMES	MICROCHIP	BREED	ALONE/NOT ALONE	SEX	AGE
Pis	380260041021944	M	NA	M	3
Schizzo	380260040995215	M	NA	M	2
Nervo	380260041021359	M	A	M	2
Sofficino Gandhi	380260040911346	M	NA	M	6
Sally	380260041021159	P	NA	F	2
Ululì	380260000947785	M	A	M	2,3
Chica	380260000947946	M	NA	F	1,3
Bugs Bunny	380260040963413	M	NA	M	1,3
Mary	380260000936843	M	NA	F	1
Ab	380260040926747	M	NA	F	4
Frollo	380260040931428	P	NA	M	1
Bistecca	380260040929898	P	A	M	1
Max	380260000941876	P	A	M	3
Ugo	380260000936564	P	A	M	5
Pedalino	380260000933489	M	NA	M	1,6
Vecchio Rex	380260000940858	P	A	M	7
Ercole	380260000945357	P	A	M	6
Pedro	380 260000929461	P	A	M	6
Benjo	380260002134065	P	NA	M	3
Pippo	380260002133164	M	NA	M	3
Margot	380260002133541	M	NA	F	3
Rott	380260002133641	P	NA	F	2
Ribes	380260002166348	M	NA	M	2
Bho	380260002171583	P	NA	M	2
Bravery	380260002177736	P	NA	F	2
Pinza	380260002185476	M	NA	M	2
Ettore	380260002167372	M	A	M	5
Peggy	380260000937386	P	NA	F	3

ers and reaction pf dogs during the the Novel Object test

CONDITIONS AT ENTRY	Symptoms	1 - Leukocytes	Neutrophils
Poor conditions. parasitic otitis and ectoparasites	None	7,87	5,89
Good	Vomiting and diarrhoea	19,10	15,8
Good	None	17,00	13,6
Good	None	6,57	4,24
Good	None	13,20	8,6
Dislocated hip-joint	Diarrhoea	11,80	9,94
Good	Diarrhoea	11,10	8,42
Good	None	17,30	15,2
Good	Diarrhoea	25,60	20,6
Good	None	13,80	9,22
Good	Diarrhoea	16,00	13,9
Good	None	11,60	8,68
Good	None	27,00	21,7
Good	Vomiting, diarrhoea and loss of appetite	12,40	9,88
Good	Diarrhoea	12,40	8,15
Good	Bilateral otitis	13,70	10,2
Good	None	15,30	13,1
Good	Diarrhoea	12,50	9,18
Good	None	17,1	10,4
Good	Inflammation of the penis	14,9	10,9
Good	None	12,9	7,5
Good	None	6.40	4.84
Good	None	28,3	23,2
Good	None	10,8	7,8
Good	None	13,3	7,78
Good	Cough reverse and bloody diarrhoea	10,5	4,85
Good	None	10,3	7,77
Good	None	11,7	6,32

ymphocyt	Monocytes	Eosinophils	Red cells	Haemoglobin	Haematocrit	Lysozyme (µg/ml)	Serum protein	Albumin/Globulin	Haptoglobin	CD4/CD8
0,837	0,603	0,46	7,02	15,6	47,3	3,53	6,80	1,00	1,37	2,05
1,31	1,12	0,584	6,74	16,7	51	6,00	7,10	0,73	1,31	2,56
1,2	1,95	0	7,43	16,1	51,9	7,28	6,40	0,85	1,32	2,06
1,19	0,913	0,181	6,81	14,4	44,8	5,58	7,70	0,62	4,38	1,60
1,76	1,65	1,08	5,85	14,1	43,4	4,99	7,20	0,59	1,52	2,01
0,843	0,815	0,139	7,19	17	50,1	7,86	6,80	0,71	2,41	0,79
0,978	1,14	0,414	7,19	16,7	50,5	7,17	7,10	1,05	1,32	1,48
0,629	1,17	0	7,07	16,8	50,9	7,93	7,00	0,75	2,15	1,90
1,54	3,18	0,013	6,38	15,7	47,9	7,67	6,50	0,82	1,31	2,98
1,87	0,747	1,86	6,87	16,6	48,4	2,23	6,10	0,93	1,61	4,13
1,02	0,975	0,034	7,37	17,9	52,8	3,11	6,40	1,04	1,54	2,85
1,7	0,729	0,459	7,14	17,7	52,6	4,55	6,40	0,88	1,10	3,59
1,52	3,15	0,634	7,66	18,8	53,6	7,68	6,90	0,81	2,23	2,10
1,46	0,903	0,013	8,52	20,3	57	3,58	7,50	0,55	2,92	3,10
2,58	0,888	0,687	7,8	18,7	54,4	2,12	6,50	0,99	1,56	4,76
1,27	1,32	0,849	7,97	19,3	56,3	6,66	8,20	0,59	2,60	1,85
1,2	0,943	0,039	8,61	20,4	58,3	2,82	6,40	1,05	1,26	2,98
1,54	0,762	0,906	6,69	15,9	46,3	3,26	7,00	0,79	0,39	1,50
2,97	1,53	2,01	6,67	15,2	46,3	3,13	7,30	0,73	1,88	2,89
1,32	1,15	1,4	7,5	18,4	52,2	2,77	6,90	0,79	0,00	3,12
3,25	1,04	1,05	6,94	17,8	51,5	2,44	6,6	0,74	1,96	1,74
0.553	0.023	0.984	7.25	17.2	53.0	2.85	7,8	0,8	9.5	4.66
1,42	2,51	0,439	6,44	16,1	46.6	1,7	6,9	0,94	2,58	2,21
1,08	1,07	0,769	7,52	18,2	52,7	2,01	6,8	0,72	10,46	2,57
1,92	1,33	2,18	7,02	16,6	49,2	2,17	6,5	0,77	7,13	3,8
2,24	1,32	1,97	8,34	21,5	60,8	1,54	6,6	1,03	1,91	1,69
0,596	1,68	0,06	7,07	17,4	51,3	2,35	7	0,93	4,14	5,9
3	0,618	1,76	7,42	19,2	55,6	13,5	6,7	0,78	1,56	2,4

**Total antioxidants**	**dRoms**	**Ehrlichia**	**Leishmania**	**2 - Leukocytes**	**Neutrophils**	**ymphocyt**	**Monocytes**	**Eosinophils**	**Red cells**
	23	N	N	12,50	8,35	1,67	1,29	1,14	7,08
		N	N	22,90	12,9	5,84	1,87	2,12	7,99
	34	N	N	13,90	9,41	1,46	2,05	0,696	7,35
	32	N	N	6,69	4,43	1,14	0,567	0,438	7,19
		N	N	14,50	10,4	2,14	1,14	0,722	6,59
377	51	N	N	9,09	7,15	0,772	0,73	0,323	7,22
295		N	N	13,80	10,8	1,57	0,944	0,442	7,24
318	64	N	N	8,68	6,58	0,772	1,19	0,001	7,29
409	41	N	N	13,20	9,12	2,2	1,67	0,005	7,55
599		N	N	8,59	4,76	1,6	1,05	1,08	6,93
665	12	N	N	23,30	18,9	1,34	2,65	0,256	6,92
417	14	N	N	7,76	4,51	2,02	0,591	0,584	7,87
476	74	N	N	11,00	5,33	2,58	1,46	1,52	7,64
699	67	N	N	14,40	11	1,9	1,4	0,002	7,99
363	16	N	N	16,20	12,4	1,87	1,23	0,733	7,86
504	26	N	N	15,60	8,49	1,83	1,42	3,61	6,47
296		N	N	13,50	9,07	1,74	1,54	1	8,01
358	49	N	N	12,80	7,75	1,86	1,29	1,8	7,21
642	33	N	N	14,5	8,4	2,65	0,647	2,75	7,69
689	45	N	N	21,1	18	1,37	1,29	0,155	7,98
405	42	N	N	10,2	5,91	2,67	0,662	0,907	8,22
491	39	N	N	9.73	6.90	1.05	1.17	0.581	7.50
302	40	N	N	14,6	10,6	1,32	1,58	0,802	7,16
312	71	N	N	13,7	7,9	2,2	0,855	2,51	7,29
306		N	N	11,9	6,94	2,51	0,816	1,51	7,37
342	16	N	N	11,2	6,42	2,21	0,805	1,66	7,73
350	48	N	N	9,28	6,42	1,24	0,737	0,788	7,76
116	9	N	N	6,03	3,65	0,502	0,284	1,6	7,3

Haemoglobin	Haematocrit	Lysozyme (µg/ml)	Serum protein	Albumin/Globulin	Haptoglobin	CD4/CD8	Total antioxidants
16	47,7	3,48	7,00	0,79	0,64	2,70	
16,7	50,1	3,07	6,30	0,72	3,95	2,00	
16	49,4	4,88	6,10	0,89	1,55	2,64	
14,8	47,4	5,58	7,40	0,75	2,14	0,77	350
15,4	48,6	7,17	6,00	0,87	0,31	3,20	260
17,2	51,1	6,72	6,90	0,79	4,76	0,69	321
16,4	50,2	8,12	6,50	0,76	4,71	1,53	309
17,1	51,7	3,59	6,50	0,72	4,88	0,60	302
19	54,2					2,16	
16,6	48,8	6,35	7,10	0,71	1,16	2,10	454
16,7	47,4	5,50	6,50	0,84	2,85	3,20	280
19,6	56,2	3,42	6,80	0,81	1,47	2,90	495
18,5	53,8	3,94	6,70	0,75	1,94	2,80	424
18,9	53,7	4,13	7,10	0,58	2,31	2,19	142
18,9	55,1	4,13	6,20	0,96	0,30	4,67	389
16	46,1	3,58	6,80	0,59	2,60	1,34	89
18,7	54	3,42	6,00	0,87	0,34	3,44	55
17,2	49,9	6,35	7,40	0,68	1,25	1,22	124
17,9	52	2,85	6,90	0,80	2,26	1,9	536
19,5	56,8	3,44	6,80	0,82	2,68	2,3	401
20,2	59,7	1,86	7,3	0,96	1,82	2,2	242
17.4	51.7	1.05	6,4	0,81	2.23	3.68	201
17,7	51,6	1.94	6,2	0,69	5,05	4,84	318
17,2	50,1	2,26	6,7	0,73	2,2	3,2	332
17,3	50,5	2,13	6,4	1,14	1,61	2,76	279
19,6	56,8	1,83	6,3	0,83	9,23	2,5	369
19	56	3,36	6,7	0,91	1,21	3,25	323
18,6	54,8	2,38	6,3	0,83	0,16	2,67	476

dRoms	Ehrlichia	Leishmania	Minutes	1. Activity	2. Aggressivity generic	3. Aggressivity tw hum	4. Aggressivity tw dogs	5. Anxiety
37	N	N	294	2,79	0,0000	0,0510	0,0034	0,9014
16	N	N	300	1,89	0,0000	0,0900	0,0100	0,5100
21	N	N	300	4,55	0,1833	0,0733	0,1067	0,9333
59	N	N	283	0,49	0,0000	0,0106	0,0000	0,3958
5	N	N	300	1,53	0,0000	0,0367	0,2533	0,4700
41	N	N	300	1,42	0,0433	0,0033	0,0533	0,4133
33	N	N	257	3,88	0,0272	0,0039	0,0039	0,7899
	N	N	287	0,21	0,0000	0,0000	0,0000	0,6551
	N	N	300	1,50	0,0067	0,0100	0,0033	0,4400
	N	N	300	2,58	0,0167	0,0133	0,0233	0,8233
55	N	N	292	2,45	0,0137	0,0000	0,0034	0,6164
	N	N	300	0,90	0,0000	0,0100	0,0100	0,7567
	N	N	300	0,75	0,0000	0,0433	0,0000	0,8133
	N	N	300	0,58	0,0000	0,0467	0,0000	0,4500
31	N	N	300	0,77	0,0000	0,0200	0,0167	0,3100
63	N	N	300	1,31	0,0000	0,0133	0,0000	0,4267
34	N	N	300	5,93	0,0000	0,0200	0,0167	0,5667
51	N	N	300	11,61	0,0000	0,0300	0,0067	0,4633
26	N	N	300	0,39	0,0100	0,0067	0,0267	0,5367
49	N	N	300	1,34	0,0100	0,0000	0,0567	1,0500
7	N	N	300	1,19	0,0133	0,0000	0,0233	0,5300
6	N	N	300	5,03	0,0200	0,0000	0,0167	0,9967
55	N	N	300	1,91	0,0200	0,0033	0,0133	0,2933
30	N	N	300	1,03	0,0033	0,0100	0,0233	0,2933
10	N	N	300	1,16	0,0167	0,0000	0,0100	0,1433
53	N	N	300	4,25	0,0367	0,0000	0,0400	1,2167
83	N	N	300	4,42	0,0400	0,0033	0,0100	0,7433
	N	N	300	1,24	0,1067	0,0567	0,0400	0,2567

5.bis Stereot norm	5.tris Att. Spost.	6. Attentiveness	7. Dom twd Hum	8. Dom twd dogs	9. Dom generic
0,0102	0,8912	6,88	0,000	0,014	0,0850
0,0267	0,4833	2,75	0,000	0,053	0,0067
0,0300	0,9033	3,95	0,290	0,237	0,2400
0,0035	0,3922	1,78	0,032	0,007	0,0283
0,0000	0,4700	5,43	0,110	0,393	0,0767
0,0033	0,4067	2,72	0,060	0,203	0,1867
0,0428	0,7043	6,78	0,342	0,311	0,2685
0,0035	0,6516	1,43	0,010	0,000	0,0035
0,0267	0,3967	5,01	0,090	0,180	0,0767
0,0467	0,6400	5,90	0,003	0,007	0,0067
0,0137	0,6027	6,23	0,000	0,301	0,0171
0,0100	0,7267	3,00	0,000	0,120	0,0067
0,1067	0,6367	5,47	0,020	0,023	0,0400
0,0100	0,4233	1,84	0,007	0,017	0,0067
0,0100	0,3000	3,46	0,043	0,050	0,0500
0,0000	0,4267	4,59	0,000	0,000	0,0033
0,0033	0,5567	6,58	0,013	0,200	0,0567
0,0033	0,4600	6,55	0,000	0,003	0,1333
0,0133	0,5233	1,69	0,017	0,037	0,0333
0,0067	1,0433	3,37	0,013	0,130	0,0767
0,0867	0,4300	2,68	0,027	0,100	0,1200
0,0000	0,9900	7,52	0,030	0,457	0,9100
0,0200	0,2233	6,77	0,160	0,097	0,6100
0,0333	0,2600	4,66	0,023	0,150	0,1233
0,0000	0,1400	4,83	0,030	0,057	0,1467
0,3200	0,8967	5,65	0,000	0,057	0,1367
0,0567	0,6733	4,55	0,017	0,053	0,3600
0,0400	0,2167	2,64	0,077	0,063	0,3467

9bis. Dom gen+twd hum	10. Subord twd human	11. Subord twd dogs	12. Subord generic	12.bis Subord gen+sub vs hum	13. Playfulness
0,0850	0,0918	0,0850	0,0167	0,1051	0,0306
0,0067	0,1600	0,2667	0,0500	0,2100	0,3667
0,5300	0,2800	0,0667	0,0071	0,2871	0,0000
0,0601	0,0707	0,0035	0,0167	0,0873	0,0000
0,1867	0,5333	0,0100	0,0033	0,5367	0,0067
0,2467	0,0633	0,0000	0,1479	0,2112	0,0000
0,6109	0,3385	0,2140	0,0070	0,3455	0,0156
0,0139	0,0627	0,0000	0,0333	0,0961	0,0000
0,1667	0,0967	0,0200	0,0000	0,0967	0,2033
0,0100	0,6800	0,0200	0,0479	0,7279	0,0000
0,0171	0,4795	0,0856	0,0067	0,4827	0,0274
0,0067	0,5800	0,0033	0,0300	0,6100	0,0000
0,0600	3,3033	0,0100	0,0200	3,3233	0,0000
0,0133	0,2233	0,0100	0,0167	0,2400	0,0000
0,0933	1,2100	0,0167	0,0000	1,2100	0,0067
0,0033	0,7133	0,0067	0,0000	0,7133	0,0000
0,0700	0,5100	0,0000	0,1267	0,6367	0,0000
0,1333	0,9967	0,0100	0,0033	1,0000	0,0000
0,0500	0,0100	0,0100	0,1200	0,1300	0,0000
0,0900	0,0267	0,0133	0,1400	0,1667	0,0000
0,1467	0,0300	0,0267	0,0467	0,0767	0,1267
0,9400	0,0033	0,0033	0,0767	0,0733	0,0000
0,7700	0,0100	0,0100	0,0433	0,0533	0,0000
0,1467	0,0033	0,0000	0,0033	0,0067	0,0000
0,1767	0,0000	0,0000	0,1567	0,1567	0,0000
0,1367	0,0333	0,0000	0,0800	0,0833	0,0000
0,3767	0,0067	0,0200	0,7500	0,7567	0,0000
0,4233	0,0733	0,0233	0,7600	0,8333	0,0000

14. Vocal communicab	15. Sociabil twd human	16. Sociabil twd dogs	17. Sociabil generic	17.bis TOT Soc gener+soc vs um	18. Quiet-laziness	
0,2279	0,2585	0,1054	0,020	0,279	0,405	
0,1600	0,0933	0,1633	0,000	0,093	0,450	
0,4433	0,0167	0,0000	0,000	0,017	0,397	
0,0565	0,0177	0,0177	0,004	0,021	0,307	
0,5433	0,0433	0,0100	0,020	0,063	0,363	
0,6567	0,0267	0,0067	0,017	0,043	0,307	
0,5798	0,0078	0,0195	0,004	0,012	0,444	
0,0557	0,0627	0,0000	0,000	0,063	0,533	
0,3133	0,1233	0,0267	0,020	0,143	0,353	
0,9433	0,7167	0,0433	0,100	0,817	0,327	
0,3082	0,4418	0,2363	0,096	0,538	0,599	
0,1733	0,1100	0,0033	0,000	0,110	0,500	
0,3633	0,1900	0,0067	0,020	0,210	0,290	
0,1567	0,0200	0,0067	0,003	0,023	0,390	
0,1700	0,0600	0,0200	0,003	0,063	0,563	
0,0333	0,0067	0,0067	0,017	0,023	0,257	
0,1300	0,1400	0,1533	0,090	0,230	0,203	
0,1967	0,0433	0,0100	0,073	0,117	0,127	
0,0233	0,0067	0,0333	0,013	0,020	0,217	
0,0733	0,1633	0,0667	0,277	0,440	0,460	
0,2467	0,0667	0,0700	0,237	0,303	0,283	
0,5233	0,0800	0,0500	0,177	0,257	0,073	
0,7000	0,1433	0,0633	0,253	0,397	0,130	
0,0600	0,0567	0,0867	0,143	0,200	0,260	
0,0400	0,1967	0,1400	0,353	0,550	0,167	
0,4933	0,0700	0,0367	0,163	0,233	0,220	
0,3167	0,0833	0,0467	0,400	0,483	0,267	
0,1400	0,0333	0,0133	0,057	0,090	0,323	

Latency approach killer whale (app less see)	REACTION (pos=1; neg=0)
1,34	0
0,02	1
0,14	1
0,02	1
0,09	1
0,00	1
0,01	1
0,01	1
0,05	1
0,01	1
18,02	0
0,01	1
0,01	1
0,03	1
28,32	0
1,21	1
1,16	1
16,47	0
0,01	1
8,54	1
0,57	1
0,00	0
0,22	0
0,00	1
2,30	0
0,09	0
0,02	1
2,40	0
