# Supplementary material for: Bold personality makes domestic dogs entering a shelter less vulnerable to diseases
Source: PLoS One. 2018 Mar 29;13(3):e0193794. doi: 10.1371/journal.pone.0193794 (PMC5875777; doi:10.1371/journal.pone.0193794)
Supplement: S2 Table — (PDF) [file pone.0193794.s002.pdf]

S2. Table with raw data regarding latency for finding the food during the the T-maze test

	Food right side										Food left side									
**Names**	1 latency finding food	Direction	2	Direction	3	Direction	4	Direction	5	Direction	6	Direction	7	Direction	8	Direction	9	Direction	10	Direction
Pis	20,00		20,00		20,00		8,46		0,17		1,02		0,19		3,55		0,26		0,50	
Schizzo	0,44		0,08		0,08		0,06		0,33		0,50		0,11		0,43		0,41		0,37	
Nervo	4,12		20,00		11,41		20,00		1,50		16,28		3,52		7,27		0,59		19,28	
Sofficino Gandhi	5,00	L	3,08	L	5,08	L	5,09	L	3,36		3,37	L	4,00	L	2,17	R	3,16		6,57	R
Sally	5,30	L	6,50	L	3,56	L	6,33	L	2,53	R	3,29	L	4,09	R	3,32	R	3,28	R	3,27	L
Ululì	3,12	R	4,19	R	10,13	R	5,42	R	2,44	R	5,02	L	2,12	R	3,25	R	5,21	L	3,34	
Chica	6,45	R	4,44	R	6,00	R	7,09	R	2,52	R	3,40	R	5,28	L	3,28	R	3,00		2,55	L
Bugs Bunny	8,32	R	4,56	L	7,27	R	6,56	R	5,33	R	5,00	R	18,36	L	3,42	L	4,04	L	3,47	L
Mary	5,54		4,40		2,47		0,31		0,28		3,43	R	1,29	R	1,50	R	1,31	L	1,20	R
Ab	20,00	L	9,38	L	4,41	L	0,41	L	0,08	R	1,30	R	0,20	R	0,28	R	0,19	R	0,19	R
Frollo	11,16	L	2,59	R	1,10	R	0,39	R	0,39	R	4,00	R	0,52	L	3,49	R	0,45	L	0,47	L
Bistecca	7,48		1,14		2,23	R	1,41	R	1,00	R	1,28	R	4,30	R	1,08	L	0,53	R	1,19	R
Max	19,48		0,45	L	1,05		0,28	R	0,36	R	8,45	R	1,50	R	0,34	L	0,34	L	0,30	L
Ugo	1,48	R	0,51	R	0,24	R	6,04	L	0,55	R	2,04	L	0,47	L	0,45	L	0,38	L	0,45	L
Pedalino	0,16	R	0,26	R	0,16	R	0,18	R	0,30	R	0,51	R	0,29	R	0,21	L	0,10	L	0,15	R
Vecchio Rex	3,12	L	4,07	L	20,00	L	17,28	R	2,58	L	3,08	L	1,47	R	0,23	L	0,28	L	0,10	L
Ercole	20,00	L	2,51	L	20,00	L	20,00		20,00	R	5,09	L	2,40	R	3,28	R	3,23	L	2,44	L
Pedro	19,40	R	20,00		6,14		20,00		4,44		12,10	R	2,49	R	5,37	R	5,00	R	8,27	L
Benjo	14,35	L	5,16	R	17,39	R	0,32	R	0,21	R	3,37	R	1,48	R	2,00	R	2,07	L	0,37	L
Pippo	20,00	L	20,00		1,35	R	2,22	R	12,13	R	2,58	L	1,26	L	0,24	L	0,58	L	1,05	L
Margot	7,15	L	1,04	R	0,36	R	1,36	L	1,12	R	1,00	R	1,35	R	0,49	R	0,47	R	0,11	L
Rott	1,14	R	20,00	Doesn't move	20,00	Doesn't move	20,00	R	5,20	R	20,00	Doesn't move	20,00	R	20,00	R	20,00	Doesn't move	20,00	Doesn't move
Ribes	0,36	R	1,58	R	6,12	R	0,25	R	0,11	R	2,40	R	0,47	R	1,01	R	0,36	R	0,12	L
Bho	7,58	L	0,57	R	0,09	R	0,34	R	0,20	R	0,13	R	2,22	R	4,00	R	0,19	R	0,35	R
Bravery	1,06	R	18,09	L	0,33	R	0,08	R	0,26	L	20,00	R	19,00	R	0,23	R	0,26	R	1,19	R
Pinza	6,25	L	0,48	R	0,11	R	0,19	R	0,56	L	0,51	R	0,49	R	0,16	R	0,09	L	0,06	L
Ettore	20,00	L	9,19	L	13,10	R	1,17	R	0,20	R	0,41	R	0,27	R	0,34	R	0,09	L	0,06	L
Peggy	0,57	R	1,16	L	1,11	R	0,09	R	0,11	R	0,22	R	0,33	R	0,48	R	1,21	R	0,35	R
